# Regulation of germ layer formation by pluripotency factors during embryogenesis

**DOI:** 10.1186/2045-3701-3-15

**Published:** 2013-03-11

**Authors:** Ying Cao

**Affiliations:** 1 Model Animal Research Center of Nanjing University and MOE Key Laboratory of Model Animals for Disease Study, 12 Xuefu Road, Pukou High-Tech Zone, Nanjing, 210061, China

**Keywords:** Pluripotency factors, Early embryogenesis, Germ layers, Cell fate decision, *Xenopus*

## Abstract

The classical pluripotency factors Oct4, Klf4, Sox2, and Nanog are required for the maintenance of pluripotency and self-renewal of embryonic stem (ES) cells and can reprogram terminally differentiated cells into a pluripotent state. Alteration in the levels of these factors in ES cells will cause differentiation into different lineages, suggesting that they are critical determinants of cell fates. These factors show dynamic expression patterns during embryogenesis, in particular in the pluripotent or multipotent cells of an early stage embryo, implying that they are involved in the cell fate decision during early embryonic development. Functions and the underlying molecular mechanisms have been extensively studied for these factors in ES cells under cultured conditions. However, this does not mean that the results also hold true for intact embryos. In the review, I have summarized and discussed the findings on the functions and the underlying mechanisms of the classical pluripotency factors during early embryogenesis, in particular during germ layer formation.

## Introduction

Pluripotency and self-renewal are two distinct features of mammalian embryonic stem (ES) cells. This means that ES cells, on one hand, are capable of differentiation into all germ layer lineages including germ cells, and on the other, can generate equivalent daughter cells. ES cells or the inner cell mass of a morula embryo express numerous genes, some of which code for transcription factors or transcriptional co-regulators. Among them, a few transcription factors, primarily Oct4, Sox2, Klf4, cMyc, Nanog, and Lin28, are required for the maintenance of pluripotency and self-renewal of ES cells [1-6]. Moreover, these factors are able to reprogram terminally differentiated somatic cells into a pluripotent state [7-12]. Manipulation of the levels of these factors in ES cells will result in differentiation toward to specific lineages [1,13-15]. Therefore, these factors play critical roles in cell fate decision. In ES cells, these factors comprise a core regulatory network for the maintenance of pluripotency and self-renewal [16,17]. Besides these “classical” pluripotency factors, more proteins have been elucidated to play a role in the maintenance of ES cell pluripotency and self-renewal, primarily via the integration to the core regulatory network, such as Tcf3, Wdr5, esBAF, Ring1A/B, Zfp296, Nr5a2, Esrrb, etc. [18-26].

The development of an animal embryo from a fertilized egg is a process of cell differentiation and movements. During vertebrate embryogenesis, the early embryonic cells are induced to differentiate into the three germ layers in a strict spatiotemporal manner. Under the regulation of inducing and patterning signals, the three germ layers will subsequently turn into all types of tissues and organs. With the progress of embryonic development, the potential of differentiation of early embryonic cells decreases gradually, i.e. the loss of pluripotency. The procedure resembles the differentiation of ES cells. Pluripotency factors are present in early cleavage embryos. It is plausible that these factors play important roles in embryonic development. Nevertheless, the functions of pluripotency factors inferred from ES cells that are cultured under artificial conditions don’t necessarily reflect their functions in intact embryos. Moreover, deletion of these genes in mouse are usually embryonic lethal and makes further functional analysis difficult in embryos. The studies with other model organisms especially *Xenopus* or zebrafish have provided profound insights into the functions of these factors during early embryogenesis. In this review, I will focus on the regulatory effects of the 'classical' pluripotency factors on early embryonic development and sum up briefly the functions of some of the ‘nonclassical’ factors.

### Oct4

**Figure 1:**
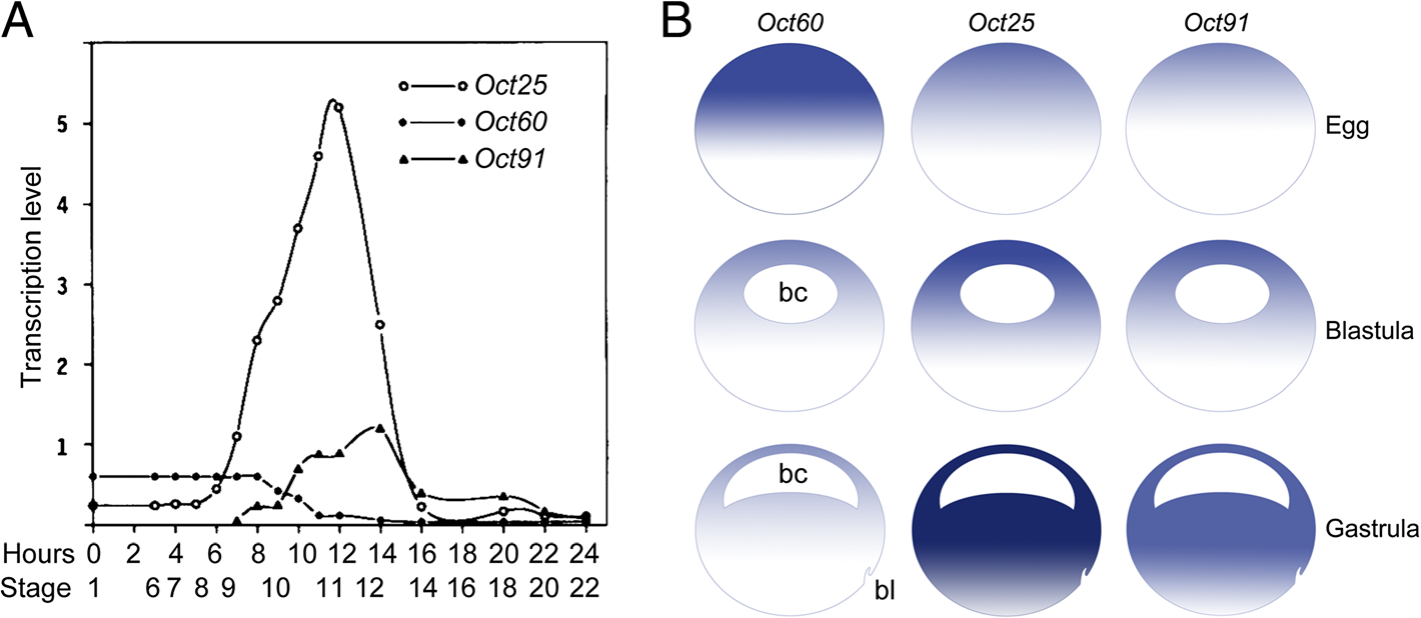
**Schematic illustration of spatiotemporal expression of *Oct60*, *Oct25 *and *Oct91 *during *Xenopus *embryogenesis.** (**A**) Temporal expression of *Oct60*, *Oct25* and *Oct91* in different stages of development. Reproduced from [30] with minor modifications. (**B**) Spatial distribution of *Oct60*, *Oct25* and *Oct91* transcripts in fertilized egg, blastula and gastrula embryos. Heavier color represents higher level of transcription. bc: blastocoel; bl: dorsal blastopore lip.

Oct4 is a transcription factor in the POU family of subclass V (POU-V). It is the key factor in the regulatory circuitry that governs pluripotency and self-renewal of ES cells. During mouse embryogenesis, *Oct4* transcript is transcribed in unfertilized oocytes. The maternal transcript is soon degraded after fertilization. The zygotic transcription shows a dynamic pattern, with specific localization to the cells of inner cell mass (ICM) of blastocyst and primitive ectoderm at implantation [27]. After day 8, its transcription in somatic cells is silenced, but it is only transcribed in primordial germ cells (PGCs) [28,29]. In *Xenopus*, there are three POU-V factors, Oct60, Oct25 and Oct91 [30-32]. However, the genes for these factors are expressed in distinct patterns during embryogenesis. *Oct60* is a maternal transcript, which is degraded soon after mid-blastula transition (MBT). The zygotic transcript is present only in very low level. *Oct25* is transcribed both maternally and zygotically, with the peak level of transcription present during gastrulation. While in the case of *Oct91*, only zygotic transcription exists [30] (Figure 1). Whole mount in situ hybridization (WISH) have demonstrated that in oocytes and cleavage stage embryos, transcripts are only detected in the animal hemispheres [33]. During blastula and gastrula stages, they are present in the animal and marginal zones [33,34] (Figure 1). At neurula stage, expression of *Oct25* is restricted to two narrow stripes within the neural plates, with one stripe in each side of the midline. This expression disappears in tailbud embryos. Instead, it is specifically expressed at the tail tip [33,34]. *Oct91* has an identical spatial expression pattern to *Oct25*. However, they differ in the levels of transcription. Using WISH, it has been not able to reveal the expression of either *Oct60*, *Oct25* or *Oct91* in the PGCs. Nevertheless, it can be displayed with RT-PCR that *Oct91*, but not *Oct60* or *Oct25*, is activated specifically in the PGCs in *Xenopus* neurula embryos, when somatic fates are committed. This suggests that among the three *Oct4* homologous genes, *Oct91* is orthologous to mammalian *Oct4*[35]. Although the spatiotemporal expression pattern of each *Xenopus POU-V* gene is different from *Oct4* in mouse embryos, the combined expression of the three *POU-V* genes corresponds to that of mouse *Oct4*. The three *POU-V* genes are also conserved in *Xenopus tropicalis*. They are arranged in a cluster and the temporal order of expression is reverse to their orientation within the cluster. Moreover, the three genes are in a syntenic region conserved between *Xenopus*, mouse and zebrafish [33,36]. They obviously arose by duplication of a common ancestor. Except *Xenopus*, only one *Oct4* homologous gene has been detected in other organisms, such as zebrafish (Pou2) [37], Axolotl [38], or chick [39]. The expression patterns of *POU-V* genes in these animals are not identical, though, the common feature is that they are expressed in the pluripotent and multipotent cells during early during early embryogenesis, and later on, in the germ cells.

A few studies have shown that *Xenopus* POU-V factors regulate the signaling cascade of germ layer induction and body axis patterning during embryogenesis. In a yeast one-hybrid screening for the factors that bind to and regulate the target gene of BMP signaling pathway (see **Glossary**), *Xvent2B*, Oct25 was identified as such a regulator [34]. It binds to *Xvent2B* promoter and interacts with Xvent2 protein, thus forming a regulatory complex on *Xvent2B* promoter. Takebayashi-Suzuki et al. [40] also reported that Oct25 regulates the competence of ectodermal cells to BMP signaling, thus playing a role in the fate choice of ectoderm. Studies by Cao et al. [36] and by Snir et al. [41] have demonstrated that *Xenopus* POU-V factors promotes neuroectoderm formation but inhibits neuronal differentiation. In addition to the regulatory effect on BMP signaling, both overexpression and knockdown experiments display that the *Xenopus* POU-V factors prevent early embryonic cells from differentiation into mesoderm and endoderm (combined as mesendoderm hereafter) germ layers [34,36]. This is in agreement with that Oct4 is responsible for the maintenance of undifferentiated state of ES cells. The function of *Xenopus* POU-V factors in embryos can be explained by that they regulate both maternal and zygotic signals being responsible for the induction of germ layer differentiation. Biochemical data demonstrate that Oct25 blocks the activities of maternal VegT and β-catenin [42], which initiate the zygotic signaling cascades for mesendoderm formation. Oct25 interacts with VegT or Tcf3, a signal transducer of Wnt/β-catenin signaling, thus form repression complexes on the promoters of VegT or β-catenin target genes [42]. Another study demonstrates that Oct4 also inhibits Wnt/β-catenin signaling in ES cells and blocks Wnt8-induced secondary axis formation (see **Glossary**) in *Xenopus* embryos [43]. Nevertheless, the underlying mechanism seems to be different since Oct4 interferes Wnt/β-catenin signaling via destabilization of β-catenin [43]. It is not known in ES cells, whether Oct4 and Tcf3 also form a repression complex and regulates Wnt/β-catenin target gene expression. One possibility accounting for the discrepancy is the intrinsic differences between signals in ES cells and *Xenopus* early embryonic cells. Nodal/Activin pathway, which is initiated by maternal VegT and β-catenin in *Xenopus* embryos, is the ultimate inducing signal for mesendoderm formation. *Xenopus* POU-V factors also show significant repression effect on Nodal/Activin activity in both whole embryos and animal caps [36,44]. Biochemical analysis reveals that there is an intrinsic connection between Oct25 and Nodal/Activin signaling. Oct25 interacts with the signal transducers of Nodal/Activin signaling, e.g. FoxH1 (FAST1), thus blocking the transcription of Nodal/Activin target genes [44]. In addition to the regulatory role in mesendoderm formation, a function in mediating early morphogenesis is also implied for *Xenopus* POU-V factors in a recent work [45]. In zebrafish, depletion of both maternal and zygotic zOct4 (also named Spiel-ohne-grenzen (Spg) or Pou2) leads to delay of epiboly (see **Glossary**) and defect in body axis patterning [46-48], resembling the effect of knockdown of POU-V in *Xenopus*[36]. In zebrafish embryos, mutation for maternal-zygotic *spg* (MZ*spg*) leads to delay of gastrulation, failure of endoderm formation and accordingly, failure of gene expression for endoderm specification. However, neuroectoderm, mesoderm and germ cells can still form in the mutant embryos [46,47]. Khan et al. [49] report that the active (VP-) or repressive (en-) form of zOct4 in zebrafish embryos exhibits different effects on mesendoderm formation. When the endogenous zOct4 activity was inhibited with an inducible, repressive form of zOct4 at different stages, different results were observed. Inhibition from midblastula stage leads to enhanced endoderm formation at the cost of mesoderm, opposite to the MZ*spg* phenotype. On the contrary, inhibition from late blastula promotes mesoderm formation at the expense of endoderm [49]. Similarly, injection of *zOct4* at different doses or repression of zOct4 activity at mid- or late blastula leads to different effects on dorsoventral patterning or convergent-extension movements [50] (see **Glossary**). These results suggest that the functions of Oct4 during embryogenesis are rather complicated. It will be interesting to investigate the effects of microinjection of zebrafish pou2 into *Xenopus* embryos. In *Oct4* null ES cells, *Xenopus* POU-V factors are able to substitute Oct4 to retain self-renewal to different degrees, in contrast, zebrafish Oct4/Pou2/Spg is not [33]. These imply that POU-V proteins in different organisms have both common and distinct functions.

In *Xenopus*, overexpression of each of the three *Xenopus* POU-V factors or mouse Oct4 generates similar effects on mesendoderm differentiation. Moreover, Oct60 and Oct25 are exchangeable in rescue experiments [36]. This raises the question which one among the three POU-V factors is the orthologue of mammalian Oct4. Two pieces of evidence support that Oct91 is more likely to be the orthologue. One is that only *Oct91* is expressed in the germline of *Xenopus* neurula embryos when somatic *Oct91* expression is silenced [35]. The other piece of evidence is that Oct4-null ES cells transfected with *Oct91* plasmid show the best rescuing effect compared to *Oct60* or *Oct25* transfection [33].

The POU-V transcription factors are characterized by a unique POU-specific domain (POUs) located at the N-terminal region and a POU homeodomain (POUh) at the C-terminal region, which are joined by a linker region [51]. The two domains are responsible for the binding of the protein to the octamer motif ATGCAAAT on the promoter of target genes, with the POUs binding to the ATGC half-site and the POUh binding to the AAAT half-site [48]. Cao et al. [52] analyzed in detail the contribution of the two domains to the function of Oct25. Overexpression of a series of deletion mutants in *Xenopus* embryos clearly showed the difference of these domains. The mutant lacking both the POUs and POUh loses its activity in regulation of germ layer formation and body axis patterning. Deletion of the POUh domain from the protein does not change its activity significantly in overexpression assays. In contrast, deletion of the complete POUs leads to a mutant that has no effect on embryogenesis. It is interesting that partial deletion, alteration of the order of a few amino acids, or point mutation of a single amino acid in the POUs results in the reversal of the protein activity. Overexpression of such mutants in embryos leads to the promotion of mesendoderm differentiation, dorsalization (see **Glossary**) of body axis and correspondingly, the upregulation of genes such as *Sox17α*, *MyoD*, *Sox2*, *Chordin*, *Goosecoid*, etc. Moreover, Oct60, Oct91 or mouse Oct4 with such mutations display similar activity to Oct25 mutants [52]. Therefore, the integrity of the POUs structure is critical for the protein function. The effect of the POUs mutant proteins on embryogenesis is also a confirmation for the function of endogenous POU-V proteins in *Xenopus* embryos. Since the molecular mechanisms underlying the function of POU-V during embryogenesis or in ES cells have been not well understood, the dominant-negative mutant might be a useful tool to elucidate such mechanisms.

**Figure 2:**
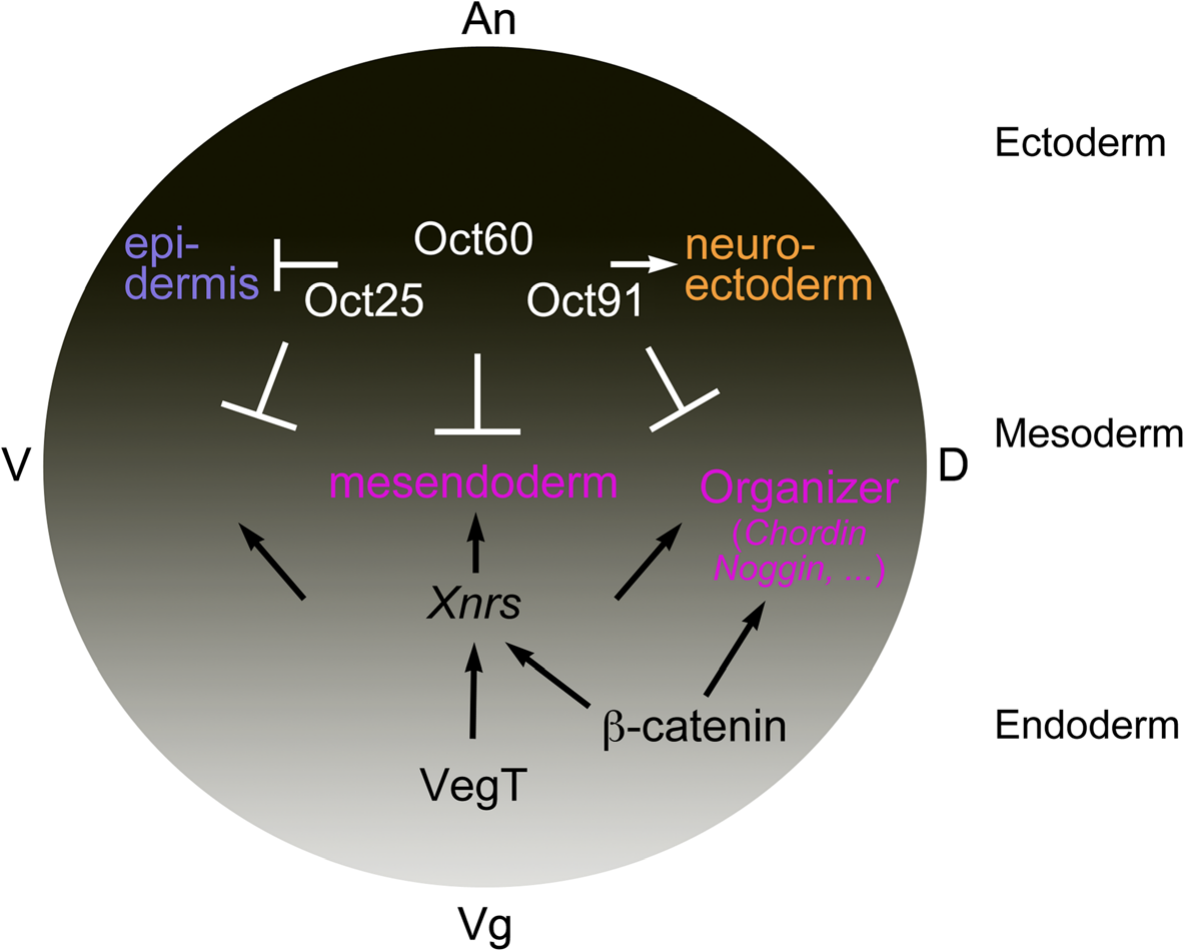
**Correlation between pluripotency factors and differentiation signals during *Xenopus *germ layer formation.** Germ layers are formed in strict spatiotemporal patterns. Either POU-V factors or germ layer inducing signals have specific localizations and exhibit different levels of activities during the process of germ layer formation of *Xenopus* embryos. POU-V factors antagonize the differentiation signals, so as to ensure that germ layer formation can occur in the correct spatiotemporal patterns (See text for details). An: animal pole; Vg: vegetal pole; D: dorsal side; V: ventral side. Reproduced from [42] with modifications.

Based on *Xenopus* study, we propose a model for the germ layer formation (Figure 2). Germ layers are formed in strict spatiotemporal patterns. The signals that maintain the undifferentiated state of early embryonic cells, typically POU-V factors, localize primarily to the animal half of embryos, where they promote neuroectoderm formation and restrict the mesendoderm inducing signals to the correct location. Maternal VegT is confined only to the vegetal region of early embryos, and overlaps with maternal β-catenin in the dorsal-vegetal cells. VegT activates ligand genes of Nodal/Activin signaling, which subsequently induces mesendoderm differentiation. β-catenin enhances transcription of Nodal/Activin ligand genes, thus establishing a Nodal/Acvtivin gradient along the dorsoventral axis. Meanwhile, it also activates signaling cascades for the formation of the organizer. There is also low abundance of *POU-V* expression in vegetal region. Here they serve to control VegT and β-catenin activities, such that differentiation of germ layers can occur correctly. Hence, the regulation of differentiation signals by the pluripotency factor POU-V establishes a balance of the two types of signals. The balance is critical for the differentiation and patterning of germ layers. Disruption of the balance will lead to the deviation of spatiotemporal patterns of normal germ layer formation.

### Nanog

Nanog was identified in a screening for the factors that maintain ES cell pluripotency and self-renewal [53] and in a screening for factors that underlie pluripotency in both ICM and ES cells independent of LIF/Stat3 [54]. During mouse embryogenesis, *Nanog* expression is restricted to pluripotent tissues such as the ICM of early blastocysts and in the epiblast of late blastocysts [53,55]. It is also specifically expressed in mouse germ cells [56]. *Nanog*-null embryos develop no epiblast or extraembryonic ectoderm [54], suggesting that Nanog is required for early embryonic development. During zebrafish embryogenesis, *Nanog* is a maternal transcript and zygotic expression persists through early cleavage to 9h post-fertilization, with peak level of transcription occurring during gastrulation [57]. Genomic analysis, database mining or screening failed to identify a *Nanog* homolog in *Xenopus*, suggesting that *Nanog* is lost in the genus during evolution [57-59], although *Nanog* orthologues have been known in other amphibians such as Axolotl [60]. The works of Schuff et al. [57] and Scerbo et al. [59] suggest that *Xenopus* might use the Vent1/2 (Ventx) transcription factors, which are known to play key roles in the ventralization of body axis [61], as alternatives to Nanog. Vent1/2 and Nanog are members of the BarH subfamily of homeodomain proteins and share some common features [57,59]. Overexpression of zebrafish Nanog (zNanog), mouse Nanog (mNanog) or *Xenopus* Vent1/2 in *Xenopus* embryos all leads to similar phenotype [57,59]. Detailed analysis demonstrates that the Vent1/2 are in fact not mere ventralizing factors. Like Nanog in mouse ES cells, Vent1/2 are also required for the maintenance of uncommitted status of early embryonic cells [59]. It is still in debate whether or not Vent1/2 and Nanog are exchangeable in function. Scerbo et al. [59] report that mNanog rescues specifically *Xenopus Vent1/2* morphant, while another study shows a different result [57]. Despite the discrepancy, both studies agree that *Vent1/2* and *Nanog* genes are related and that Ventx1/2 and mNanog serve comparable functions during embryogenesis.

A few studies including the above-mentioned ones focus on the functional homology of Nanog or Nanog-like proteins in different species [39,57,59,60]. Nevertheless, the function of Nanog during germ layer formation has been less understood. The only existing data don’t agree well. One typical study shows that zNanog regulates endoderm formation via direct activation of the transcription of the homeobox domain factor Mxtx2, which in turn specifies the yolk syncytial layer that emits signals for endoderm induction [62]. However, overexpression of mNanog, zNanog or Vent1/2 in *Xenopus* embryos results in the inhibition of expression of endoderm marker genes [57,59]. Moreover, most mesoderm and ectoderm marker genes are also downregulated [57,59]. The disparity might be due to the difference in signals regulating germ layer formation in *Xenopus* and zebrafish, or due to the difference in experimental setup. Nevertheless, more efforts are required to characterize the functions of Nanog during embryogenesis.

### Klf4

**Figure 3:**
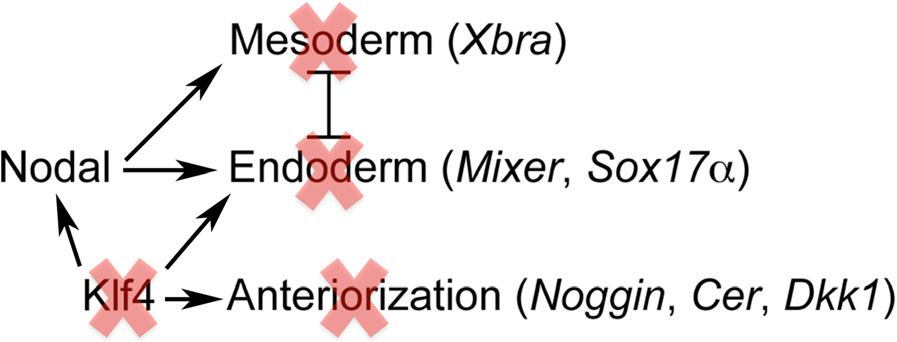
**A model for the function of *Klf4 *during *Xenopus *germ layer formation.** Klf4 induces endoderm differentiation via activation of Nodal pathway or directly via activation of endoderm inducing genes, which repress mesodermal gene expression. Klf4 also promotes dorsalization of body axis via stimulation of a subset of genes in the Spemann organizer. When Klf4 activity is blocked, germ layer formation and body axis patterning are also inhibited.

Klf4 is a member in the family of Kruppel-like factors (Klf). All these transcription factors contain three highly conserved classical Cys2/His2 zinc-fingers at the carboxyl terminus and transcriptional regulatory domain at the amino terminus of the protein. The zinc-fingers are responsible for the binding to the CACCC-box in the DNA. Klf family consists of 17 members (Klf1-Klf17), which are involved in many biological processes such as proliferation, apoptosis, differentiation and development [63,64]. The clue that Klf4 is a pluripotency factor is manifested by the induced pluripotent stem cells (iPS) with defined factors including Klf4 [7]. It is also a component of the regulatory circuitry required for the maintenance of pluripotency and self-renewal [22]. In agreement with this function, *Klf4* is transcribed in ES cells [15]. During mouse embryogenesis, *Klf4* transcript is present in embryos as early as at E4.5 (late blastocyst), and the transcription persists in later stages of development [65]. It is not clear whether *Klf4* transcription is localized, for example, to the ICM, or is ubiquitous in blastocyst, nor is it not known whether *Klf4* is transcribed during earlier stages of mouse embryogenesis. Microarray data suggest that there is little maternal *Klf4* transcript in mammals [66]. In contrast, *Klf4* was identified as maternal transcript in fish and frog [67,68]. This might be due to the divergence of gene regulation between lower and high vertebrates during evolution. Zygotic transcription was also observed during early embryogenesis of fish and frog [67,68]. It seems that Klf4 in mice does not participate in germ layer formation or body axis patterning during early embryogenesis, because *Klf4*^−/−^ mice just show the failure in establishing proper skin barrier function, which eventually leads to the death of embryos shortly after birth [69]. It has been reported that zebrafish Klf4 is involved in mesendoderm differentiation, hatching, and erythropoiesis [70,71]. However, it is worthwhile to mention here that some reported expression patterns or functions of zebrafish Klf4 could have been assigned improperly [70-72]. Re-examination of the sequence of zebrafish *Klf4* used in these studies (Accession number: AF392994) displayed that the protein encoded by the gene might not be the genuine orthologue of mammalian Klf4. Another different sequence in the database (Accession number: NM_001113483) shows much bigger homology to mammalian Klf4. Comparison of the expression patterns of *Klf4* in different organisms in these studies also reveals the uncertainty of a true Klf4 orthologue [70-72]. Moreover, the function of zebrafish ‘Klf4’ in erythropoiesis is reminiscent of the function of Neptune, or Klf17, in *Xenopus*[73]. A similar case is that a *Xenopus* cDNA in database (Accession number: NM_001086359) is mistakenly assigned as *Klf4* since the amino acid sequence encoded by the cDNA has the highest similarity to *Xenopus* Neptune [68]. Recently, Cao and colleagues [68] describe the identification of *Klf4* orthologue in *Xenopus* (Accession number: JN126325) and investigation of its functions during germ layer formation and body axis patterning. During *Xenopus* embryogenesis, *Klf4* is transcribed both maternally and zygotically. The transcript is ubiquitous but differs in abundance in different regions of gastrulating embryos [68]. Gain of Klf4 function in *Xenopus* embryos leads to ectopic induction of endoderm differentiation in ectoderm, and the induction is achieved via Nodal-dependent and independent manners. In addition, Klf4 overexpression results in anteriorization of body axis, and the anteriorization is even stronger when either Wnt, Nodal, BMP or FGF signaling is blocked, suggesting that it also plays a role in the regulation of body axis patterning. The underlying mechanism is that Klf4 stimulates the transcription of a subset of genes in the Spemann organizer, such as *Noggin*, *Cerberus* and *Dkk1*, which code for antagonists against BMP, Wnt or Nodal. On the other hand, loss of Klf4 function causes failure of germ layer differentiation and defects in body axis formation. Correspondingly, early embryonic cells lose the responsiveness to the activity of inducing signals such as Nodal/Activin, and the genes involved in body axis patterning fail to express [68]. It seems that Klf4 is a competence factor for germ layer differentiation and body axis patterning in *Xenopus* embryos. Nevertheless, the cross-talks between Klf4 and the signals promoting germ layer differentiation and body axis patterning remain a question for further investigations. A model depicting the function of Klf4 during *Xenopus* germ layer formation and body axis patterning is shown in Figure 3.

### Sox2

Sox2 is a member of subgroup SoxB1 in the Sox (SRY-related HMG box) family of transcription factors [74]. The sequence and expression are well conserved during evolution. The most prominent feature of *Sox2* expression in different animals is the localization of transcription to neuroectoderm during gastrulation, to the neural plates during neurulation and to the central nervous system in later stages of development. Therefore, it has been used as a marker gene for neural differentiation in *Xenopus* embryos. In mouse, *Sox2* mRNA is first present in some cells at morula stages and specifically localizes to the ICM in blastocysts [2], implying that it is involved in the fate decision of early embryonic cells. *Sox2*-null mouse embryos are lethal at implantation. The embryos show the lack of epiblast but expansion of extraembryonic ectoderm, suggesting that it is essential for epiblast cell identity [2]. However, when both maternal and zygotic *Sox2* mRNA is removed using RNAi, mouse embryos arrest at morula stage and trophectoderm cannot form [75]. Both the results above demonstrate that *Sox2* is indispensible for early development, and blocking either maternal or zygotic *Sox2* generates different effects on the differentiation of early embryonic cells. In *Xenopus*, there is relatively low abundance of *Sox2* maternal transcript, but zygotic transcription increases dramatically since midblastula transition [76], implying that it is involved in the stage of development, when germ layer induction is initiated. It seems to be difficult to detect the spatial distribution of *Sox2* transcript in *Xenopus* embryos earlier than gastrulation using whole mount in situ hybridization [77,78]. Therefore, much attention has been paid so far to the function of *Sox2* in neural development and little care has been taken about its roles in earlier events during *Xenopus* embryogenesis. In *Xenopus*, Sox2 alone could not induce neural differentiation, but it serves to render the ectoderm competent to the neuralizing signal FGF [77]. Further analysis demonstrates that Sox2 is required for the maintenance rather than initiation of neural differentiation [79]. The effect of blocking Sox2 activity on germ layer formation has yet not known in *Xenopus* embryos. During zebrafish embryogenesis, morpholino knockdown of either one of the zebrafish B1 Sox, i.e. Sox1, Sox2, Sox3 or Sox19, generates no effect on embryonic development, probably due to functional redundancy. However, if they are simultaneously knocked down, severe defects occur in embryos [80]. The embryos show not only defect in neural development, but also defect in dorsoventral patterning and gastrulation movements [80]. Thus, the B1 Sox factors should play a role in germ layer formation and patterning. Other studies demonstrate that inhibition of Sox3 function alone in *Xenopus* or zebrafish embryos already causes malformation of mesendoderm, probably via the regulation of Nodal and Wnt/β-catenin signaling [81-84]. Considering that Sox2 and Sox3 are close relatives, it is possible that Sox2 functions similarly to Sox3 [84]. Nevertheless, further evidence is required for confirming such a conjecture.

### Other factors

In the above, how the ‘classical’ pluripotency factors regulate early embryonic development is discussed. I would also like to mention in passing the functions of other pluripotency-related factors during early embryogenesis, such as Tcf3, Wdr5, esBAF, Ring1A/B, Zfp296, Nr5a2, Esrrb, etc. [18-26]. Tcf3 is a key signal transducer for Wnt/β-catenin signaling pathway, but it is a transcription repressor in absence of Wnt activation. It has been well established that Tcf3 plays an essential role in germ layer formation and dorsoventral (anteroposterior) body axis patterning during early embryonic development of *Xenopus* and other animals [85-88]. Wdr5 and Ring1A/B are involved in the regulation of *Hox* genes, thereby mediating anteroposterior axis formation, but they seem to have no effect on germ layer formation [89,90]. So far, there are no records for the functions of esBAF or Zfp296 during early embryogenesis, except for a few reports about their involvement in the pluripotency regulatory network [20,23,91,92]. Esrrb and Nr5a2 are nuclear receptors and have been extensively explored for their functions in differentiation, metabolism and diseases, etc. They are also identified as regulators of pluripotency in recent years and can act as substitutes of Oct4, Klf4 or Nanog in reprogramming or in maintenance of pluripotency [24-26,93]. So far, there are no data about its function in germ layer formation or body axis patterning. In fact, during *Xenopus* or zebrafish embryogenesis, *Nr5a2* (also named *Lrh1*, *Ftz-f1*) is not present in early cleavage and gastrula embryos [94,95]. This expression pattern rules out the possibility of the involvement in the early events of embryonic development, for example, germ layer induction and pattern formation. Similarly, *Esrrb* (or named *ERRβ*, *errβ*) is only detected in zebrafish embryos at 20hpf, when germ layer formation and body axis patterning are completed [96]. There exist two other related genes *ERRα* and *ERRγ*. The former is possibly involved in early events of embryogenesis because of its expression from cleavage to gastrula embryos of zebrafish, but the latter is also expressed much later [96]. The temporal expression patterns of these genes during zebrafish embryogenesis are somewhat similar to those in mouse. Inactivation of *errβ* in mouse results in lethal placental defects [97], but knockout of *errα* does not affect embryonic development significantly [98]. Therefore, the functions of most of these ‘nonclassical’ pluripotency factors during early embryogenesis remain to be elucidated.

## Conclusions and perspectives

Here I summarize the discovery of the functions of the 'classical' pluripotency factors during early embryonic development, especially germ layer formation. These factors, Oct4, Nanog, Klf4 and Sox2, comprise of a regulatory network for the maintenance of pluripotency and self-renewal, and reprogram terminally differentiated cells into a pluripotent state. However, they play different functions during germ layer formation via distinct regulatory mechanisms in *Xenopus* embryos. Meanwhile Oct4 homologous proteins inhibit the differentiation potential in early embryonic cells, and Klf4 promotes the tendency of differentiation. At the level of molecular mechanisms, the former block the activity of the signals that promote differentiation, while the latter does the opposite, as discussed above. Although it is not clear how Sox2 functions during germ layer differentiation, some hints imply that it might be also a repressor of such an event, because Sox2 can rescue Oct25 morphant in which differentiation is enhanced, while Klf4 can not (personal observation, unpublished data). Functional differences between these pluripotency factors are also manifested in ES cells or iPS cells. Knockout of one of these genes in ES cells leads to differentiation towards different lineages. Oct4 and Sox2 are prerequisite and play the major roles for the iPS, while Klf4 and Nanog play the supporting roles. Such differences are critical for the fine-tuning of signals such that they reach to a balanced state in the cells. During embryogenesis, this means that pluripotency factors like Oct4 serve to regulate the differentiation signals thereby the signals will operate in correct locations and right time, and at the exact levels. Disruption of the balance will lead to the spatiotemporal aberrations of differentiation of early embryonic cells, hence the failure of germ layer formation and body axis patterning. Although the studies as mentioned here have enlightened us of many aspects of the function of pluripotency factors during embryogenesis, however, much more work is required to resolve the discrepancies and get an unbiased understanding of their roles in cell fate decision during germ layer formation and body axis patterning.

### Glossary

**BMP signaling pathway:** The bone morphogenetic protein signaling pathway. BMP ligands are a subgroup of TGFβ family of secreted growth factors. The signaling is activated upon the binding of the ligand to the type II transmembrane receptor, which subsequently phosporylates type I receptor. Afterwards the receptor I activates receptor-specific Smad transducers (R-Smads), namely Smad1, 5 and 8, by phosphorylation. The activated R-Smad interacts with the co-Smad, Smad4, and the complex translocates into nucleus, where they recruit specific transcription factors to the promoters of target genes and regulate target gene transcription. Studies on *Xenopus* embryogenesis have demonstrated that BMP signaling pathway plays crucial roles in dorsoventral body axis patterning and neural induction. BMP signaling is responsible for the establishment of ventral structures of *Xenopus* embryos and neural induction requires inhibition of BMP activity.

**Convergent extension movements:** a process of cellular rearrangements and migration by which an early embryo is formed into a long and narrow body shape during embryogenesis. During gastrulation, cell intercalation cause the narrowing of one direction (convergence) and the elongation of a perpendicular direction (extension), thus establishing the body plan of an animal. Convergent extension movements also occur during organogenesis to generate an elongated pattern, for example, the gut. Convergent extension movements are well studied in *Xenopus* embryogenesis, however, it is believed that convergent extension movements are conserved in invertebrates and vertebrates.

**Dorsalization:** the body plan of an animal embryo is composed of dorsal types of tissues, such as the central nervous system and the notochord, and ventral type of tissues, such as blood, along the dorsoventral axis. The formation of dorsal type of tissues, or dorsalization, and the formation of ventral type of tissues, or ventralization, is the dorsoventral patterning of germ layers during gastrulation.

**Epiboly:** usually it refers to the movement of ectodermal cells during gastrulation by which ectoderm spreads to cover the whole of the embryo. It is a coordinated process of cell division, shape change and cell intercalation of the ectoderm, which becomes thinner and then wraps the deeper layers of the embryo.

**Secondary axis formation:** the generation of a second body plan in addition to the primary one in a vertebrate embryo. The secondary axis formation was first demonstrated by Hans Spemann and Hilde Mangold in 1924 in a graft experiment in which they transplanted a small piece of tissue, the dorsal blastopore lip, of one newt (donor) gastrula embryo into the opposite side (ventral side) of the dorsal blastopore lip of another (host) newt gastrula embryo. The grafted dorsal lip was able to ‘organize’ the neighboring cells of the host embryo to form a complete secondary dorsal-ventral and antero-posterior pattern of body axis in addition to the primary body axis, and the graft itself became the notochord. As a result, the host embryo developed a Siamese twin. The Nobel Prize winning (1935) experiment first established the crucial importance of cell-cell inductions during embryonic development. Now it has been recognized via studies in *Xenopus* embryonic development that the dorsal lip, or called the Spemann-Mangold organizer, is a rich source of secreted factors, such as Noggin, Chordin, Dkk1, Cerberus and so on, that antagonize the activities of the growth factors being emanated from the ventral cells, including BMPs, Wnts and Nodal. Ventral expression of these secreted factors or activation of maternal Wnt pathway can mimic the effect of the dorsal lip graft experiment and induce the formation of secondary axis in the embryos.

## Competing interests

The author declares that he has no competing interests.
